# Assessment of abduction motion in patients with rotator cuff tears: an analysis based on inertial sensors

**DOI:** 10.1186/s12891-019-2987-0

**Published:** 2019-12-12

**Authors:** Cristina Roldán-Jiménez, Miguel Cuadros-Romero, Paul Bennett, Steven McPhail, Graham K. Kerr, Antonio I. Cuesta-Vargas, Jaime Martin-Martin

**Affiliations:** 1 Department of Psychiatry and Physiotherapy, University of Malaga, Faculty of Health Sciences, Arquitecto Francisco Peñalosa 3, Campus de Teatinos, 29071 Málaga, Spain; 2 Clinimetric Group F-14 Biomedical Research Institute of Malaga, (IBIMA), Málaga, Spain; 3 Unit of Upper Limb Orthopedic Surgery of Hospital at University of Malaga, Málaga, Spain; 4 Institute of Health & Biomedical Innovation, Faculty of Health, Queensland University Technology, Brisbane, Australia; 5 Centre for Functioning and Health Research, Metro South Health, Brisbane, Australia; 6 Department of Human Anatomy, Legal Medicine and History of Science. Legal Medicine Area, University of Malaga, Faculty of Medicine, Malaga, Spain

**Keywords:** Kinetic, Upper extremity, Shoulder, Rotator cuff

## Abstract

**Background:**

Reduced range of motion in the shoulder can be a source of functional limitation. The use of inertial sensors to quantify movement in addition to more common clinical assessments of the shoulder may allow clinicians to understand that they are potentially unnoticed by visual identification. The aim of this study was to generate an explanatory model for shoulder abduction based on data from inertial sensors.

**Method:**

A cross-sectional study was carried out to generate an explanatory model of shoulder abduction based on data from inertial sensors. Shoulder abduction of thirteen older adults suffering from shoulder dysfunction was recorded with two inertial sensors placed on the humerus and scapula. Movement variables (maximum angular mobility, angular peak of velocity, peak of acceleration) were used to explain the functionality of the upper limb assessed using the Upper Limb Functional Index (ULFI). The abduction movement of the shoulder was explained by six variables related to the mobility of the shoulder joint complex. A multivariate analysis of variance (MANOVA) was used to explain the results obtained on the functionality of the upper limb.

**Results:**

The MANOVA model based on angular mobility explained 69% of the variance of the ULFI value (r-squared = 0.69). The most relevant variables were the abduction-adduction of the humerus and the medial/lateral rotation of the scapula.

**Conclusions:**

The method used in the present study reveals the potential importance of the analysis of the scapular and humeral movements for comprehensive evaluation of the upper limb. Further research should include a wider sample and may seek to use this assessment technique in a range of potential clinical applications.

## Background

Shoulder disorders are highly prevalent in the population and to a greater extent among older adults; in many cases, biomechanical anomalies are asymptomatic [1, 2]. Despite the potential absence of pain, limitations caused by a reduced range of motion (ROM) can have a detrimental impact on the performance of activities of daily living [3]. Clinically, the evaluation of patients’ daily functioning in their upper limbs is often based on self-administered questionnaires, such as Disability of the Arm Shoulder and Hand (DASH) [4] or Upper Limb Functional Index (ULFI) [5] and functional physical assessments [6]; due to the high cost of the imaging devices such as motion tracking with multiple camera system or real-time ultrasound.

In this regard, there is increasing evidence supporting the combined use of new technologies with functional physical tests or ROM assessments for more comprehensive biomechanical diagnostics. Technologies used for this purpose include X-ray [7–9], magnetic resonance imaging [10], 3D imaging models [11] and inertial sensors [12, 13], among others. Currently, one of the devices most frequently used for clinical purposes is inertial sensors due to their small size, reliability, and accuracy for registering human movement (speed, acceleration, and orientation) [14].

For the assessment of shoulder mobility with inertial sensors, it is necessary to use two or three inertial sensors located on the skin adjacent to the humerus, scapula, and chest [15]. Inertial sensors have been used alongside other objective assessments of the quality and quantity of movement in patients with damaged shoulders [16, 17]. Similarly, these sensors have sufficient sensitivity to discriminate between healthy and affected subjects, complementing the results of standardized assessment scales [16, 17].

Changes in shoulder mobility, whether symptomatic or asymptomatic, may be due to the bone and muscle-related disorders affecting the rotator cuff [8–11, 18]. This may limit upper limb functionality and, in some cases, is directly associated with age [18]. Prior research has indicated there are no significant differences in the neutral positioning of the shoulder complex (esternoclavicular, scapulothoracic, humerothoracic, acromioclavicular) in symptomatic and asymptomatic subjects. However, differences in glenohumeral kinematics exist between asymptomatic and symptomatic subjects. In individuals with damaged shoulders, glenohumeral angular positions are limited to elevation of the arm, coinciding with a reduction in scapulothoracic upward rotation. Significant changes in internal mobility of the shoulder joint complex have been detected when the shoulder flexes above 90 degrees [13]. This is because the scapula has different movement and muscle activation: elevation with superior fibres trapezius and elevator scapulae; depression with lower fibres trapezius; abduction with serratus major and pectoral minor muscles; adduction trapezius and romboids; external rotation with serratus anterior and internal rotation with lower fibers trapezius and rhomboids muscles [19], which can change the orientation of the arm [20]. In addition to the scapula, shoulder muscles also change their activation level according to the speed and ROM of flexion or abduction [21].

Positive correlations between acceleration values measured with inertial sensors and function-related questionnaire responses (DASH) have been reported previously. Higher acceleration values reflected greater shoulder functionality [17]. The asymmetry of shoulder movement assessed with inertial sensors has also been correlated with patient-reported functionality; greater asymmetry indicated less functionality [22].

Therefore, describing an approach for assessing shoulder mobility based on inertial sensors in subjects who have functional limitations is an important next step in advancing the field. The assessment with multiple inertial sensors will discriminate whether the displacement of body segments is related to the functional limitation; providing additional information for clinical use. In this way, inertial sensors could be used to complement more traditional assessments when diagnosing shoulder dysfunction.

Therefore, the main objective of this study was to design a multivariate model for upper-limb dysfunction based on inertial sensors, thereby obtaining predictors of upper limb dysfunction based on shoulder movements.

## Methods

### Subjects

A cross-sectional study was designed to evaluate the abduction movement of the shoulder with two IntertiaCube3 Sensors [23]. Thirteen participants (9 females, 4 males) were recruited from a specialized orthopedics clinic. They had previously been diagnosed with partial rotator cuff tears by magnetic resonance imaging and were on the waiting list for surgical intervention. Inclusion criteria were age between 18 and 75 years old, Body Mass Index (BMI) between 18 and 42 and presence of a confirmed rotator cuff tear. Participants were excluded if they declined to participate in the study or had concurrent or alternative etiologies for their shoulder dysfunction.

A sample size of 9 participants was calculated for an α error of 0.05, a statistical power of 0.8, based on data from a systematic review on the use of inertial sensors to measure human movement [24].

The study was approved by the ethics committee of the Research Commission of the Faculty of Health Sciences of the University of Malaga and complied with the principles of the Declaration of Helsinki [25]. All participants accepted and signed written informed consent prior to participation in the study. Informed consent contains the participant’s personal data, the purpose of the study, study protocol, anonymous treatment of the data obtained and the abandonment of the study if requested.

### Apparatus

Two IntertiaCube3 Sensors wireless systems (Billerica,MA,US) [23] were used to measure shoulder abduction of each subject. This sensor has high performance: 4 ms latency, 180 Hz rate, high accuracy (below 1° of error), 3 degrees of freedom (Yaw, Pitch, and Roll), full angular range, maximum angular rate of 1200° per second and a little size and weight. Acceleration (m/s^2^) and angular mobility (°) of shoulder abduction were measured with these sensors in the three spatial axes according to Euler angles (Z-Yaw, Y – Pitch, X - Roll); each spatial axis was related to the movement of the corporal segment (Table 1). According to the International Society of Biomechanics (ISB) [26] for the humerus, roll axis is related to flexion/extension movement, pitch axis with internal/external rotation, yaw axis with abduction/adduction movement. In the scapula, X-axis with medial/lateral rotation, Y-axis with pro/retraction and Z-axis is related to anterior-posterior-tilt.

**Table 1:** Movement sensor and ISB movement

Region	Axis- plane Sensor	ISB Movement
Humerus	X -Roll	FL–EX
	Y - Pitch	IN–EX rotation
	Z - Yaw	AB–AD
Scapula	X - Roll	ME–LA rotation
	Y - Pitch	PR-RE
	Z - Yaw	A–P tilt

*FL* Flexion, *EX* Extension, *IN* Internal, *EX* External, *AB* Abduction, *AD* Aduction, *A-P* Anterior-posterior tilt, *PR-RE* Pro/retraction, *ME-LA* Medial/lateral rotation

**Fig. 1:**
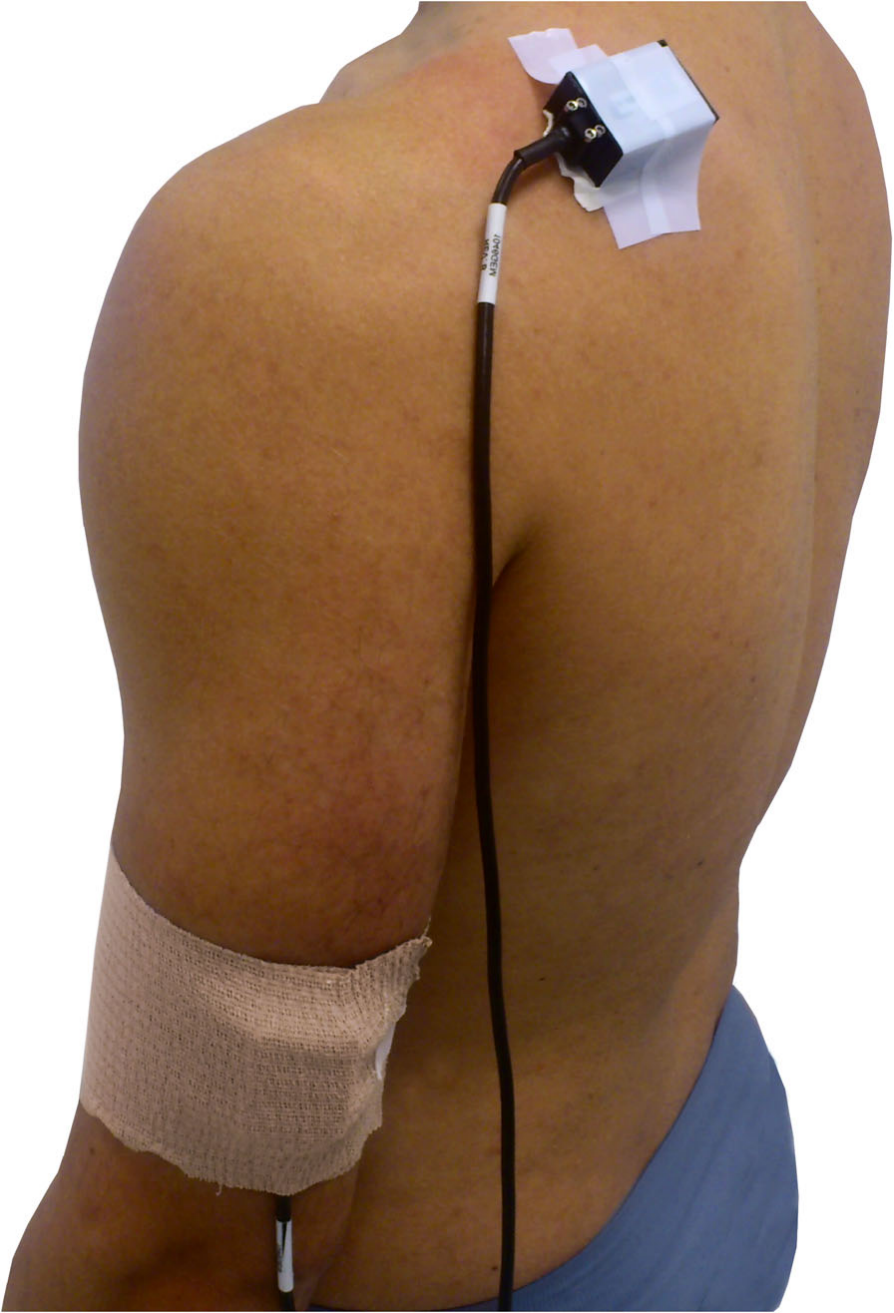
Inertial sensors placement

Two inertial sensors were placed on the humerus and the scapula following the protocol designed by Cutti et al. [15]. Scapula sensor was placed with the Z-axis in line with the cranial edge of the scapular spine over the central third of the scapula. Humerus sensor was placed over the central third of the humerus, slightly posterior. To ensure the correct measurement data, the skin of participants was cleaned with alcohol before attaching the sensors to the skin with double-sided adhesive. An elastic cohesive bandage was used on humerus, and an adhesive bandage was used on scapula (see Fig. 1).

Before making the recordings, inertial sensors were calibrated to 0 following the protocol established by the manufacturer’s software [27]. This software was the same as used for recording data; a low-pass filter (Kalman filter) was applied while recording data.

### Procedure

Prior to registration made with inertial sensors, patient characteristics including the Spanish version of Upper Limb Functional Index (ULFI) [5] were measured. ULFI is an upper extremity outcome measure that consists of a 25-item scale that can be transferred to a 100-point scale. It also has strong psychometric properties [28]. Its Spanish version has demonstrated high internal consistency (α = 0.94) and reliability (r = 0.93) [5]. Placed in the standing position and with the upper extremity in the neutral position, participants performed three full shoulder abductions. That is, lifting the arm sideways until the hand reaches as high as possible. Prior to measurements, participants performed shoulder abduction to ensure they had understood the task and they could move the upper limb without any perturbation. Two sets of three repetitions were recorded; the second repetition of each set was the one chosen to be analyzed. Patients were told to perform shoulder abduction at a natural speed until their movement had reached its end of range of motion. Questionnaires were recorded. Abduction of the affected shoulder was measured on the humeral and scapular sections. The main variables analyzed were: maximum angular mobility (°), angular peak of velocity (°/s), peak of acceleration (m/s^2^).

### Data analysis

Descriptive analyses (mean, SD) were used for participant characteristic variables (weight, height, BMI, and age) to describe the sample. Based on the yaw, pitch and roll values obtained by the sensor the minimum peaks obtained by each sensor were subtracted from the maximum peaks for each of the variables (acceleration and speed); the norm of the resultant vector (Nrv) was calculated *Nrv =* $\sqrt{x^2+{y}^2+{z}^2}$ in order to obtain the mean speed and acceleration of the movement performed. Descriptive analysis with confidence interval was made for the Nrv. The means of peak of maximum angular mobility inside the abduction movement in each of the 3 axes (yaw, pitch, and roll) were used in order to create a multivariate analysis model (MANOVA) explaining the ULFI questionnaire results. MANOVA analysis is applied if there are more two or more dependent variables, this allows to identify the effects of the variables between them individually and together. Dependent variables have theoretical relationships between them.

## Results

The functional impairment of the participants was reflected by high ULFI values (mean ± SD) 70.96 ± 20.93. The anthropometric characteristics of the participants were age 52.68 ± 9.78 years, weight 75.58 ± 17.98 kg, height 1.64 ± 0.09 m and body mass index 28.22 ± 6.59 kg/m^2^. The mean (95%CI) peak of acceleration (m/s^2^) and velocity (°/s) and peak from norm of the resultant vector in abduction are presented in Table 2.

**Table 2:** Mean (95%CI) peak of acceleration (m/s^2^) and velocity (°/s) and peak from norm of the resultant vector in ABD

Axis	Humerus	Scapula
Nrv		
Acceleration	16.25 (9.86–222.64)	4.48 (2.72–6.24)
Velocity	109.42 (70.80–147.98)	43.71 (27.97–59.45)

*Nrv* Norm of the resultant vector.

The mean maximum angular mobility in the humerus abduction axis (35.72°) indicated that a substantial reduction in shoulder mobility was present in the sample. The reductions of the range movement, also affect to scapular section, where the mean range was 12.63° for protraction-retraction (Table 3).

**Table 3:** Mean (95%*CI*) sensor peak maximum of angular mobility (°) from each axis in abduction movement

Surface placement	Humerus		Scapula	
Axis	Motion	Mean (95%*IC*)	Motion	Mean (95%*IC*)
*X*	FL-EX	38.0 (16.17–59.85)	ME-LA rotation	4.57 (1.87–7.28)
*Y*	IN-EX	77.56 (48.18–106.92)	PR-RE	12.63 (5.56–19.70)
*Z*	AB-AD	35.72 (21.12–50.31)	A–P tilt	15.60 (2.33–13.86)

*FL* Flexion, *EX* Extension, *IN* Internal, *EX* External, *AB* Abduction, *AD* Aduction, *A-P* Anterior-posterior tilt, *PR-RE* Pro/retraction, *ME-LA* Medial/lateral rotation

The MANOVA model explained 69% of the variance of the ULFI value. In an exploratory decomposition of the multivariate model, the main explanatory variable was the value of the humerus AB-AD movement (*p* = 0.093) followed by the scapular ME-LA (*p* = 0.195). However, given the limited sample size in the present study, none of these individual variables were statistically significant in the decomposition model on their own. On the other hand, there was less (or no) indication that gender and scapular anterior-posterior tiling movement was likely to have importance for the explanation of the model (Table 4).

**Table 4:** Decomposition of the multivariate model

Model	*R*	*R Square*		*Adjusted R Square*	*Std. Error of the Estimate*	
1	0.831	0.690		0.569	16.38757	
		*Unstandardized Coefficients*		*Standardized Coefficients*	*t*	*p*
		*B*	*Std. Error*	*Beta*		
1	(Constant)	108.320	9.569		11.320	0.000
	Gender	−3.189	8.920	−0.062	−.357	0.725
	FL–EX	−0.195	0.185	−0.295	−1.057	0.305
	AB–AD	−0.347	0.196	−0.590	−1.771	0.093
	IN–EX	0.104	0.154	0.192	0.673	0.510
	PR-RE	−0.078	0.058	−0.212	−1.347	0.195
	A-P tilt	−0.312	0.569	−0.123	−0.548	0.590
	ME-LA rotation	−0.119	1.072	−0.039	−0.111	0.913

*FL* Flexion, *EX* Extension, *IN* Internal, *EX* External, *AB* Abduction, *AD* Aduction, *A-P* Anterior-posterior tilt, *PR-RE* Pro/retraction, *ME-LA* Medial/lateral rotation

## Discussion

Shoulder abduction has been described by the use of two inertial sensors in the present study. In this case, they were placed on the scapula and humerus. The results obtained were used in order to create a multivariate model for the description of the abduction movement(s) that explained shoulder function as reported by patients using the ULFI questionnaire. Thus, the overall objective of the study was fulfilled. The maximum values of acceleration and angular velocity refer to the normal movement performed by the participants (they were asked to perform shoulder abduction at normal speed). This requirement allowed us to create a model that was more faithful to shoulder movement in daily living. In accordance with the results obtained, the inertial sensors have potential for complementary description and quantification of perceived disability in the upper limb; because of the results of MANOVA model (r-squared = 0.69).

According to findings from the movement decomposition model reported in Table 3, humerus ABD-ADD and scapula ME-LA rotation may be the movement components that have the greatest association with self-reported upper limb function. This finding was not surprising; however, this was accompanied in greater measure for the scapular anterior-posterior tilt movement rather than scapular ME-LA rotation. These findings from the present study are consistent with a previous study that focused on the scapulothoracic joint which found a reduced ME-LA rotation motion during elevation in symptomatic subjects [12]. In contrast with these results, another previous study that examined 3D scapula kinematics using Polhemus Fastrak found an increased scapular lateral rotation as a compensatory pattern in pathological shoulders [29]. A potential explanation for this observation is that the middle deltoid is the muscle that performs arm elevation with greater activity over 75° of shoulder abduction, while the supraspinatus is more effective at low angles [11]. Likewise, Duc et al. (2014) observed different levels of muscle activation for shoulder abduction in the same subject depending on the affected or healthy side [30].

In this regard, the rotator cuff has an important role in shoulder abduction [9]. Prior studies have shown tears in the rotator may or may not be symptomatic or associated with functional deficits, and they are positively associated with older age [10]. Several studies have demonstrated that shoulder mobility does not maintain a direct relationship with the size or thickness of the tear [10, 18]; and in some cases, there may be accommodation of the humeral head in the glenoid [8, 9, 11].

In the present study, the findings presented in the Table 4 MANOVA model may represent something of the accommodation or biomechanical adaptation of the scapula and humerus movement using the two additional planes of movement rather than abduction alone. This is consistent with prior reports from other authors regarding the 3-dimensional movement of the scapula during the performance of shoulder abduction in symptomatic and asymptomatic subjects [31]. Although the 3-dimensional movement of the scapula has been discussed in prior research, the symptomatic nature of participants in the present study (mean ULFI 70.96) and quantification of 3-dimensional movement using a straight-forward inertial sensor setup means that finding from the present study are likely to have particular relevance for this clinical population.

The association between the dysfunctionality of the upper limb (high values in ULFI) and movement of the arm was consistent with other studies. Jolles et al. (2011) identified a positive coefficient of correlation (R > 0.61) for 3 kinematic variables in 4 differents questionnaire functionality. One of the questionnaires tested, the Simple Shoulder Test, obtained an excellent linear correlation (R = 0.80) with shoulder power [17]. The results obtained by Körver et al. (2014) also showed positive relationships between kinematics asymmetry scores, understood as relative difference between healthy and affected side, and DASH and Simple Shoulder Test Questionnaires (R = 0.79); which indicated a high diagnostic power to differentiate between healthy and affected side. These results are consistent with those obtained in the present study in which the values of humeral AB-AD explained almost 60% on the negative direction of the variance of the questionnaire functionality (Table 4). Therefore, a lower level of AB-AD was associated with a higher score obtained on the ULFI (which corresponds to less functionality).

In clinical contexts, shoulder assessment is usually done by traditional clinical tests that are based on the premise that it is possible to isolate individual structures by compressing or stretching the tissue of interest. However, this is not possible without affecting the state of adjacent structures [32] because rotator cuff tendons are interwoven as a functional unit [33]. For example, some clinical tests that are intended to implicate supraspinatus pathology have been demonstrated (using electromyography) to activate eight or nine other muscles [34]. Hence, the employment of these tests leads to inaccuracy in their findings [32–35].

Furthermore, the clinical expression of shoulder injuries is highly variable [36–38]. Besides the clinical test, in shoulder assessment image tests are also employed. However, these too may be considered invalid at times, as there are a large number of asymptomatic individuals who have structural shoulder abnormalities [32]. Hence, at present, the evaluation and diagnosis of joint pathology of the shoulder joint is a complex clinical endeavor prone to uncertainty [33]. Results from the present study reinforce the potential use of both inertial sensors and questionnaires to assess shoulder function building on prior research in the field that has established each of these as independently validated measurement instruments. Research contributions that identify potential predictors of upper limb dysfunction based on validated instruments may not only have a role in diagnostics but also have additional potential in quantifying the effect of treatment. By extension, this may also lead to further developments that assist in improving predictions of which patients are likely to receive the greatest benefit from surgical or conservative interventions. In this line, the present multivariate model could be employed to shoulder assessment along the time or after treatment. For example, shoulder healing after surgery is of great interest to determine which surgical repair techniques are the best for tendon repair [39] or to maintain its integrity [40]. Furthermore, it is important to determine the effect of post-surgical rehabilitation [41–43], and current research is focused on clinical outcomes including both PRO and ROM [44, 45]. In this regard, future studies with a wider sample should include sensitivity and specificity assessment in order to determine its value as a diagnostic tool. To build on findings from the present study, future research may also seek to measure the same movement in a person twice (one on each side) and also for patients to report the functionality of each of their upper limbs, as well as their global upper limb functions. A study of this nature would have the potential to correlate the functional capacity of each of the sides with the same kinematics values, while also considering the roles of unilateral or bilateral kinematic deficits and hand dominance on self-reported upper limb functioning to investigate the potential mediating role of the unaffected arm.

Prior descriptions of shoulder abduction have been carried out by various authors in recent decades [7–11, 18]. Most of these prior studies focused on rotator-cuff fatigue although some elements of biomechanical mobility during shoulder abduction have been examined Unfortunately these previously reported approaches to examining biomechanical shoulder abduction movement.

### Study limitations

The inertial sensors used in the present study allowed an assessment based on angles, speed and acceleration movements. However, they do not provide the same level of insight pertaining to osseous structures involved in the movement as X-rays [7–9], ultrasound techniques [18], magnetic resonance [10] or computed tomography [11]. In the present study, the contralateral shoulder side of the participants as controls was not evaluated. Nonetheless, the supplementary biomechanical information provided by inertial sensors may add value in the context of biomechanical diagnostics in clinical settings. Furthermore, the aforementioned imaging technologies are likely to be too costly for daily use in clinical settings and the inability to follow the movement in real-time, while inertial sensors do not have these limitations [24]. However, this technique may require additional validation with different patients with shoulder pathologies before reaching more solid conclusions, this would allow their inclusion in routine clinical practice.

## Conclusions

The functionality of the shoulder is a key element in the activities of daily living. The method used in the present study reveals the potential importance of the analysis of the scapular and humeral movements for comprehensive evaluation of the upper limb. The individual analysis of the planes of movement demonstrated the importance of considering the relative contribution of each joint movement. The use of wireless 3-dimensional sensors permitted the consideration of shoulder abduction as a combination of movements dependent on each other within the joint complex of the shoulder. Future studies should be carried out with different pathologies of the shoulder comparing the affected shoulder with the healthy one as a control. Likewise, a system validation process based on blind evaluators could be carried out to determine the level of shoulder alteration, based on the values obtained by the inertial sensors. In the same way, further research may seek to use this assessment technique in a range of potential clinical applications.

## Data Availability

The datasets used and/or analysed during the current study are available from the corresponding author on reasonable request.

## Abbreviations

AB: Abduction
AD: Aduction
A-P: Anterior-posterior tilt
BMI: Body Mass Index
DASH: Disability of the Arm Shoulder and Hand
EX: Extension
EX: External
FL: Flexion
IN: Internal
MANOVA: Multivariate analysis of variance
ME-LA: Medial/lateral rotation
Nrv: Norm of the resultant vector
PR-RE: Pro/retraction
ROM: Range of Motion
ULFI: Upper Limb Functional Index
